# Supplementary material for: Geo-demographic trends in nontraumatic subarachnoid hemorrhage-related mortality among older adults in the United States, 1999–2020
Source: Front Neurol. 2024 Aug 14;15:1385128. doi: 10.3389/fneur.2024.1385128 (PMC11349650; doi:10.3389/fneur.2024.1385128)

eTable 1. Number of Nontraumatic Subarachnoid Hemorrhage Related Deaths by Sex and Race and Ethnicity in the United States, 1999-2020

eTable 2. Number of Nontraumatic Subarachnoid Hemorrhage Related Deaths by Place of Death in the United States, 1999-2020

eTable 3. Nontraumatic Subarachnoid Hemorrhage Mortality as Underlying Cause of Death among Older Adults in the United States, 1999-2020

eTable 4. Number of Nontraumatic Subarachnoid Hemorrhage Related Deaths by Region in the United States, 1999-2020

eFigure 1. Trends in ntSAH-Related Crude Mortality Rates in Older Adults Stratified by Ten-Year Age Group

eFigure 2. Trends in ntSAH-Related Deaths in Older Adults Stratified by Location

eFigure 3. Trends in ntSAH Age-Adjusted Mortality Rates as the Underlying Cause of Death in Older Adults

eTable 1. Number of Nontraumatic Subarachnoid Hemorrhage Related Deaths by Sex and Race and Ethnicity in the United States, 1999-2020

|  | Overall | Women | Men | NH White | NH Black or African American | Hispanic or Latino | Asian or Pacifc Islander | Population |
| --- | --- | --- | --- | --- | --- | --- | --- | --- |
| Year | Deaths | Deaths | Deaths | Deaths | Deaths | Deaths | Deaths | Total |
| 1999 | 3457 | 2444 | 1013 | 2823 | 286 | 210 | 122 | 34797841 |
| 2000 | 3463 | 2420 | 1043 | 2799 | 338 | 179 | 132 | 34991753 |
| 2001 | 3217 | 2224 | 993 | 2568 | 297 | 179 | 148 | 35290291 |
| 2002 | 3418 | 2386 | 1032 | 2771 | 280 | 176 | 161 | 35522207 |
| 2003 | 3300 | 2286 | 1014 | 2627 | 295 | 186 | 159 | 35863529 |
| 2004 | 3326 | 2311 | 1015 | 2665 | 258 | 240 | 144 | 36203319 |
| 2005 | 3099 | 2116 | 983 | 2466 | 271 | 219 | 129 | 36649798 |
| 2006 | 3242 | 2186 | 1056 | 2559 | 281 | 244 | 144 | 37164107 |
| 2007 | 3190 | 2148 | 1042 | 2510 | 280 | 225 | 160 | 37825711 |
| 2008 | 3230 | 2148 | 1082 | 2535 | 280 | 233 | 159 | 38777621 |
| 2009 | 3157 | 2087 | 1070 | 2488 | 278 | 208 | 165 | 39623175 |
| 2010 | 3199 | 2112 | 1087 | 2459 | 278 | 264 | 179 | 40267984 |
| 2011 | 3315 | 2161 | 1154 | 2614 | 265 | 247 | 169 | 41394141 |
| 2012 | 3398 | 2215 | 1183 | 2595 | 301 | 285 | 187 | 43145356 |
| 2013 | 3450 | 2220 | 1230 | 2640 | 285 | 296 | 207 | 44704074 |
| 2014 | 3402 | 2231 | 1171 | 2648 | 277 | 262 | 189 | 46243211 |
| 2015 | 3722 | 2415 | 1307 | 2813 | 316 | 353 | 219 | 47760852 |
| 2016 | 3877 | 2469 | 1408 | 2880 | 367 | 364 | 233 | 49244195 |
| 2017 | 4043 | 2542 | 1501 | 2996 | 408 | 337 | 262 | 50858679 |
| 2018 | 4187 | 2664 | 1523 | 3101 | 383 | 411 | 259 | 52431193 |
| 2019 | 4319 | 2734 | 1585 | 3173 | 416 | 418 | 278 | 54058263 |
| 2020 | 4633 | 2840 | 1793 | 3421 | 433 | 427 | 313 | 55659365 |
| Total | 77644 | 51359 | 26285 | 60151 | 6873 | 5963 | 4118 | 928476665 |

NH: Non-Hispanic

eTable 2. Trends in Nontraumatic Subarachnoid Hemorrhage Related Deaths by Place of Death in the United States, 1999-2020

|  | Medical Facility | Nursing home/Long term care | Hospice Facility | Home | Other/Unknown |
| --- | --- | --- | --- | --- | --- |
| Year | Deaths | Deaths | Deaths | Deaths | Deaths |
| 1999 | 2963 | 321 | NA | 127 | 36 |
| 2000 | 2896 | 381 | NA | 133 | 52 |
| 2001 | 2741 | 289 | NA | 140 | 47 |
| 2002 | 2848 | 363 | NA | 127 | 76 |
| 2003 | 2708 | 344 | NA | 161 | 67 |
| 2004 | 2715 | 361 | 13 | 145 | 83 |
| 2005 | 2503 | 345 | 42 | 139 | 69 |
| 2006 | 2587 | 344 | 64 | 156 | 82 |
| 2007 | 2548 | 342 | 80 | 146 | 67 |
| 2008 | 2527 | 310 | 109 | 166 | 70 |
| 2009 | 2383 | 351 | 118 | 168 | 64 |
| 2010 | 2448 | 335 | 157 | 184 | 70 |
| 2011 | 2516 | 343 | 173 | 202 | 76 |
| 2012 | 2529 | 322 | 237 | 217 | 83 |
| 2013 | 2501 | 357 | 256 | 230 | 96 |
| 2014 | 2401 | 374 | 292 | 264 | 68 |
| 2015 | 2650 | 381 | 350 | 279 | 61 |
| 2016 | 2736 | 394 | 359 | 330 | 54 |
| 2017 | 2793 | 439 | 390 | 351 | 65 |
| 2018 | 2952 | 406 | 388 | 360 | 76 |
| 2019 | 3018 | 444 | 413 | 356 | 85 |
| 2020 | 3088 | 458 | 445 | 530 | 111 |
| Total | 59051 | 8004 | 3886 | 4911 | 1558 |

eTable 3: Nontraumatic Subarachnoid Hemorrhage Mortality as Underlying Cause of Death among Older Adults in the United States, 1999-2020

| Year | Deaths | Crude Rates | Age-Adjusted Rates |
| --- | --- | --- | --- |
| 1999 | 2832 | 8.14 | 8.18 |
| 2000 | 2828 | 8.08 | 8.10 |
| 2001 | 2740 | 7.76 | 7.76 |
| 2002 | 2756 | 7.76 | 7.77 |
| 2003 | 2642 | 7.37 | 7.34 |
| 2004 | 2653 | 7.33 | 7.26 |
| 2005 | 2649 | 7.23 | 7.18 |
| 2006 | 2568 | 6.91 | 6.86 |
| 2007 | 2546 | 6.73 | 6.64 |
| 2008 | 2542 | 6.56 | 6.49 |
| 2009 | 2501 | 6.31 | 6.31 |
| 2010 | 2445 | 6.07 | 6.07 |
| 2011 | 2541 | 6.14 | 6.13 |
| 2012 | 2579 | 5.98 | 6.07 |
| 2013 | 2613 | 5.85 | 5.91 |
| 2014 | 2565 | 5.55 | 5.64 |
| 2015 | 2808 | 5.88 | 5.98 |
| 2016 | 2823 | 5.73 | 5.86 |
| 2017 | 2918 | 5.74 | 5.88 |
| 2018 | 3010 | 5.74 | 5.91 |
| 2019 | 3006 | 5.56 | 5.73 |
| 2020 | 3188 | 5.73 | 5.96 |
| Total | 59753 | 6.44 | 6.49 |

eTable 4. Trends in Nontraumatic Subarachnoid Hemorrhage Related Deaths by Region in the United States, 1999-2020

| Census Region | Year | Population | Deaths | Crude Rates | Age-Adjusted Rates |
| --- | --- | --- | --- | --- | --- |
| Northeast | 1999 | 699 | 7344915 | 9.52 | 9.48 |
| Northeast | 2000 | 708 | 7372282 | 9.6 | 9.53 |
| Northeast | 2001 | 706 | 7378899 | 9.57 | 9.44 |
| Northeast | 2002 | 697 | 7383537 | 9.44 | 9.29 |
| Northeast | 2003 | 704 | 7395250 | 9.52 | 9.36 |
| Northeast | 2004 | 692 | 7398832 | 9.35 | 9.1 |
| Northeast | 2005 | 653 | 7405757 | 8.82 | 8.53 |
| Northeast | 2006 | 640 | 7424583 | 8.62 | 8.33 |
| Northeast | 2007 | 680 | 7496226 | 9.07 | 8.79 |
| Northeast | 2008 | 623 | 7627994 | 8.17 | 7.82 |
| Northeast | 2009 | 653 | 7728004 | 8.45 | 8.19 |
| Northeast | 2010 | 672 | 7804833 | 8.61 | 8.35 |
| Northeast | 2011 | 643 | 7940826 | 8.1 | 7.79 |
| Northeast | 2012 | 647 | 8219446 | 7.87 | 7.73 |
| Northeast | 2013 | 684 | 8445821 | 8.1 | 7.96 |
| Northeast | 2014 | 666 | 8645194 | 7.7 | 7.62 |
| Northeast | 2015 | 719 | 8849104 | 8.13 | 8.07 |
| Northeast | 2016 | 709 | 9051592 | 7.83 | 7.82 |
| Northeast | 2017 | 711 | 9368697 | 7.59 | 7.55 |
| Northeast | 2018 | 768 | 9567017 | 8.03 | 8.08 |
| Northeast | 2019 | 736 | 9815833 | 7.5 | 7.62 |
| Northeast | 2020 | 799 | 10049658 | 7.95 | 8 |
| Midwest | 1999 | 847 | 8247014 | 10.27 | 10.23 |
| Midwest | 2000 | 830 | 8259075 | 10.05 | 9.97 |
| Midwest | 2001 | 776 | 8281265 | 9.37 | 9.25 |
| Midwest | 2002 | 800 | 8292655 | 9.65 | 9.5 |
| Midwest | 2003 | 778 | 8335947 | 9.33 | 9.18 |
| Midwest | 2004 | 766 | 8376310 | 9.14 | 8.94 |
| Midwest | 2005 | 706 | 8423434 | 8.38 | 8.21 |
| Midwest | 2006 | 760 | 8505528 | 8.94 | 8.71 |
| Midwest | 2007 | 734 | 8623362 | 8.51 | 8.3 |
| Midwest | 2008 | 767 | 8788844 | 8.73 | 8.56 |
| Midwest | 2009 | 715 | 8919777 | 8.02 | 7.84 |
| Midwest | 2010 | 724 | 9022334 | 8.02 | 7.85 |
| Midwest | 2011 | 802 | 9193520 | 8.72 | 8.54 |
| Midwest | 2012 | 741 | 9513646 | 7.79 | 7.76 |
| Midwest | 2013 | 781 | 9791313 | 7.98 | 7.9 |
| Midwest | 2014 | 813 | 10060441 | 8.08 | 8.06 |
| Midwest | 2015 | 819 | 10322425 | 7.93 | 8.04 |
| Midwest | 2016 | 867 | 10586634 | 8.19 | 8.3 |
| Midwest | 2017 | 880 | 10928244 | 8.05 | 8.15 |
| Midwest | 2018 | 914 | 11238075 | 8.13 | 8.33 |
| Midwest | 2019 | 910 | 11552973 | 7.88 | 8.1 |
| Midwest | 2020 | 1038 | 11855295 | 8.76 | 9.05 |
| West | 1999 | 714 | 6849329 | 10.42 | 10.54 |
| West | 2000 | 743 | 6922129 | 10.73 | 10.79 |
| West | 2001 | 652 | 7046947 | 9.25 | 9.32 |
| West | 2002 | 745 | 7145411 | 10.43 | 10.43 |
| West | 2003 | 719 | 7267743 | 9.89 | 9.9 |
| West | 2004 | 728 | 7384634 | 9.86 | 9.86 |
| West | 2005 | 638 | 7536487 | 8.47 | 8.46 |
| West | 2006 | 669 | 7682437 | 8.71 | 8.68 |
| West | 2007 | 671 | 7864136 | 8.53 | 8.52 |
| West | 2008 | 701 | 8110149 | 8.64 | 8.69 |
| West | 2009 | 648 | 8359024 | 7.75 | 7.75 |
| West | 2010 | 685 | 8546832 | 8.01 | 8.04 |
| West | 2011 | 677 | 8882196 | 7.62 | 7.66 |
| West | 2012 | 813 | 9321734 | 8.72 | 8.82 |
| West | 2013 | 753 | 9739904 | 7.73 | 7.94 |
| West | 2014 | 743 | 10169622 | 7.31 | 7.5 |
| West | 2015 | 781 | 10593700 | 7.37 | 7.58 |
| West | 2016 | 866 | 10984765 | 7.88 | 8.15 |
| West | 2017 | 924 | 11333408 | 8.15 | 8.45 |
| West | 2018 | 951 | 11751812 | 8.09 | 8.41 |
| West | 2019 | 1000 | 12160735 | 8.22 | 8.57 |
| West | 2020 | 1047 | 12548867 | 8.34 | 8.72 |
| South | 1999 | 1197 | 12356583 | 9.69 | 9.78 |
| South | 2000 | 1182 | 12438267 | 9.5 | 9.6 |
| South | 2001 | 1083 | 12583180 | 8.61 | 8.73 |
| South | 2002 | 1176 | 12700604 | 9.26 | 9.36 |
| South | 2003 | 1099 | 12864589 | 8.54 | 8.63 |
| South | 2004 | 1140 | 13043543 | 8.74 | 8.84 |
| South | 2005 | 1102 | 13284120 | 8.3 | 8.39 |
| South | 2006 | 1173 | 13551559 | 8.66 | 8.77 |
| South | 2007 | 1105 | 13841987 | 7.98 | 8.08 |
| South | 2008 | 1139 | 14250634 | 7.99 | 8.07 |
| South | 2009 | 1141 | 14616370 | 7.81 | 7.96 |
| South | 2010 | 1118 | 14893985 | 7.51 | 7.66 |
| South | 2011 | 1193 | 15377599 | 7.76 | 7.92 |
| South | 2012 | 1197 | 16090530 | 7.44 | 7.63 |
| South | 2013 | 1232 | 16727036 | 7.37 | 7.63 |
| South | 2014 | 1180 | 17367954 | 6.79 | 7.09 |
| South | 2015 | 1403 | 17995623 | 7.8 | 8.15 |
| South | 2016 | 1435 | 18621204 | 7.71 | 7.99 |
| South | 2017 | 1528 | 19228330 | 7.95 | 8.22 |
| South | 2018 | 1554 | 19874289 | 7.82 | 8.11 |
| South | 2019 | 1673 | 20528722 | 8.15 | 8.45 |
| South | 2020 | 1749 | 21205545 | 8.25 | 8.65 |

eFigure 1.


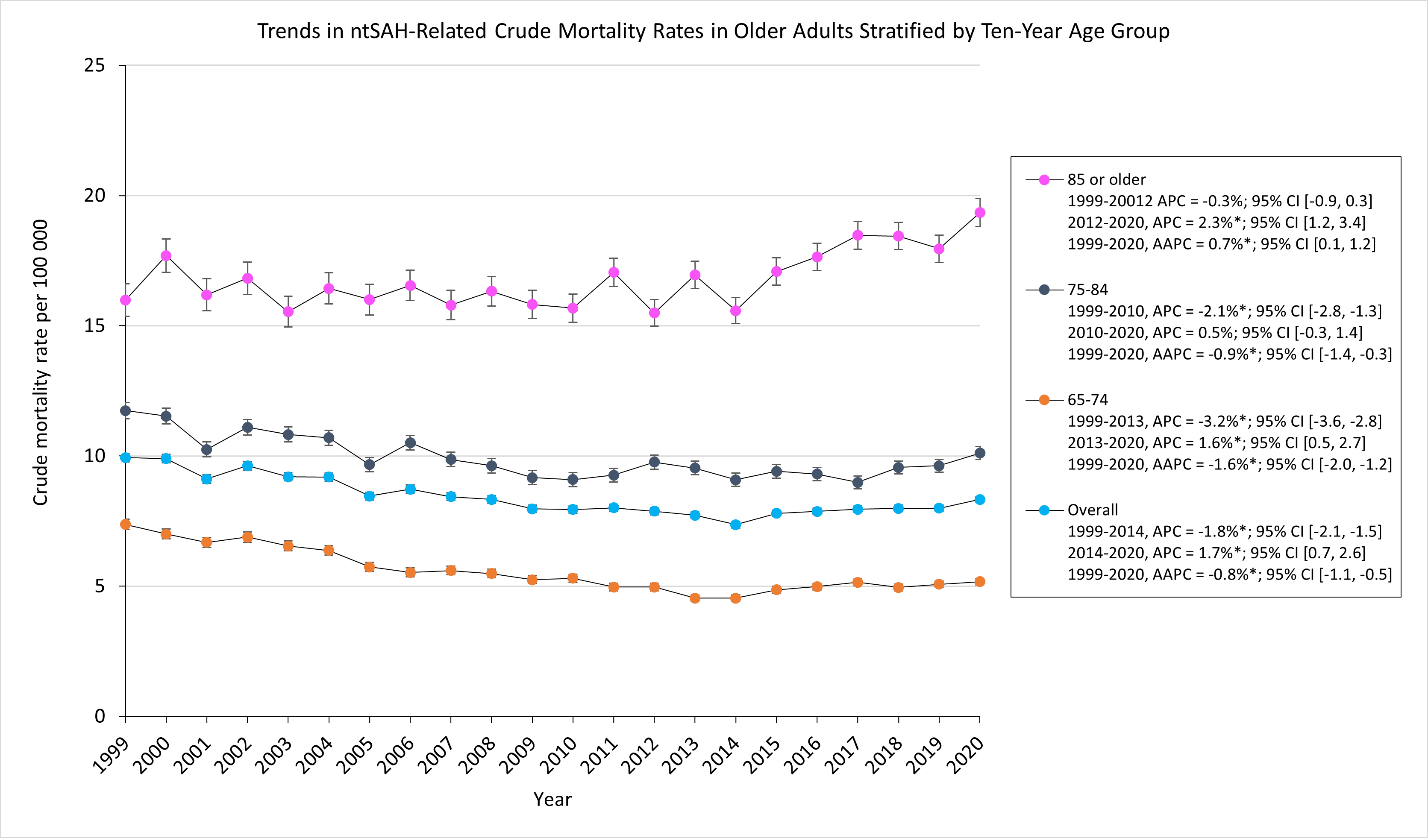


eFigure 2.


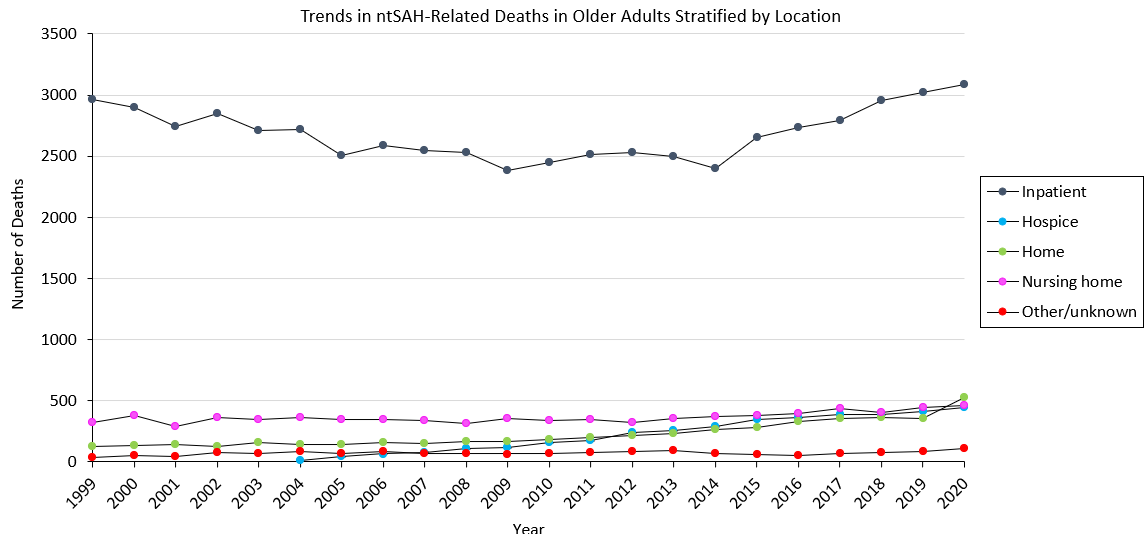


eFigure 3.


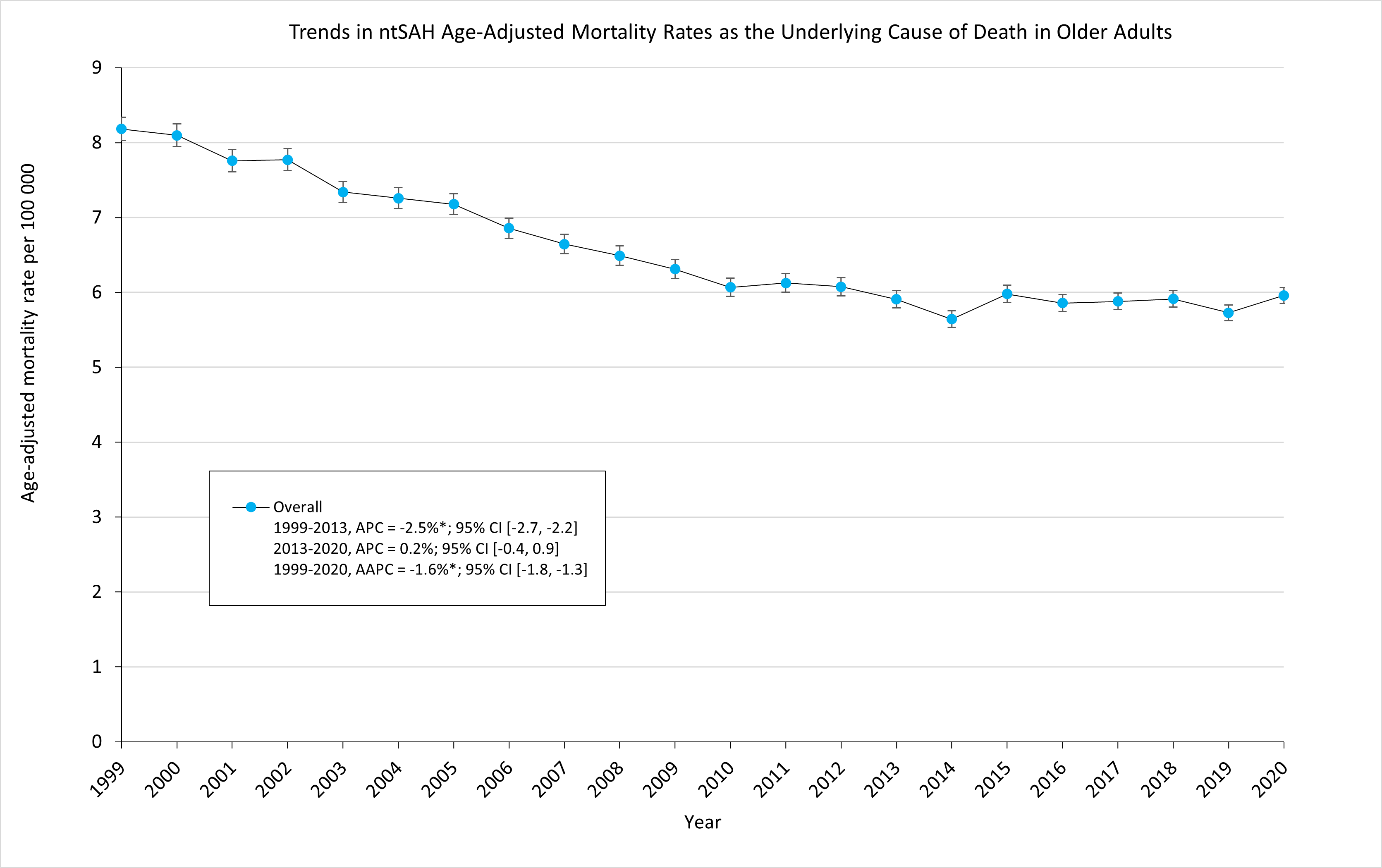

Supplement: Supplementary file 1 [file Data_Sheet_1.docx]
